# Intensive care unit dignified care: Persian translation and psychometric evaluation

**DOI:** 10.1002/nop2.2238

**Published:** 2024-07-08

**Authors:** Amir Jalali, Niloufar Darvishi, Parnia Kalhory, Fateme Merati, Salam Vatandost, Khalil Moradi

**Affiliations:** ^1^ Substance Abuse Prevention Research Center, Research Institute for Health Kermanshah University of Medical Sciences Kermanshah Iran; ^2^ Student Research Committee Kermanshah University of Medical Sciences Kermanshah Iran; ^3^ Department of Emergency and Critical Care Nursing, School of Nursing and Midwifery Kermanshah University of Medical Sciences Kermanshah Iran; ^4^ Clinical Care Research Center, Institute for Health Development Kurdistan University of Medical Sciences Sanandaj Iran

**Keywords:** dignity, intensive care unit, nursing, patient care, reliability, validity

## Abstract

**Aim:**

The present study aimed to evaluate the psychometric properties of the Persian version of the ‘Intensive Care Unit Dignified Care Questionnaire (IDCQ)’ among Iranian nurses.

**Design:**

A methodological and psychometric study was conducted in 2022, involving nurses from six teaching hospitals in Kermanshah, Western Iran.

**Methods:**

The IDCQ was translated into Persian using a forward‐backward translation method. Construct validity was assessed through exploratory factor analysis (EFA) and confirmatory factor analysis (CFA), employing a stratified sampling method with 455 critical care nurses. Internal consistency was gauged using Cronbach's alpha coefficient, while reliability was determined through the test–retest method. Analyses were performed using SPSS version 26 and Lisrel version 8 software.

**Results:**

EFA and CFA validated the instrument's two‐factor, 17‐item structure. The CFA indicated a well‐fitting model with fit indices: CFI = 0.93, NNFI = 0.92, GFI = 0.861, RMSEA = 0.051 and SRMR = 0.046. Pearson's correlation coefficient substantiated a significant relationship between the items, subscales and the overall scale. The instrument's reliability was confirmed by a Cronbach's *α* coefficient of 0.88 and a test–retest reliability of 0.86.

**Conclusion:**

The Persian version of the IDCQ, comprising two factors and 17 items, has been validated as a reliable and applicable tool for use within the Iranian nursing community.

## 1 INTRODUCTION

Nurses' crucial role in patient care necessitates adherence to professional standards, including the fundamental principle of treating patients with dignity, as outlined in ethical codes (Matiti, 2020; Vaismoradi et al., 2020). Dignity encompasses concepts such as empathy, respect and consideration for patients' values, and is influenced by human values and cultural factors (Liang et al., 2022; Lindwall & Lohne, 2021). Providing dignified care involves empathetic treatment, respect for independence and recognition of the patient's values and beliefs (Rahi, 2017; Tehranineshat et al., 2020). Therefore, it is crucial for nurses in this field to consistently prioritize their patients' dignity during interactions (Kwame & Petrucka, 2021; Sæteren & Nåden, 2021). Nurses' dignified care can significantly enhance the patient satisfaction and treatment adherence and reduce tension levels, underscoring the vital importance of dignity in nursing practice (Stephen Ekpenyong et al., 2021; Tehranineshat et al., 2020). Conversely, undignified care can cause patient stress, dissatisfaction and feelings of worthlessness (Downe et al., 2018; Lindwall & Lohne, 2021; Söderman et al., 2020). The preservation of human dignity is particularly paramount in intensive care units, where the critical nature of patients' conditions often precludes their involvement in decision‐making (Bidabadi et al., 2019; Nyholm & Koskinen, 2017).

Standardized instruments, such as the Climate of Respect Evaluation–Intensive Care Unit (CORE‐ICU), Direct Observation Checklist (DOC) and ICU Dignified Care Questionnaire (IDCQ), are essential for evaluating how nurses uphold patient dignity in intensive care units (Beach et al., 2018; Carrese et al., 2017; Liang et al., 2022). While the CORE‐ICU instrument captures the clinical staff's perspectives on ICU treatment, it does not specifically address the distinct role of nurses (Beach et al., 2018). The DOC, a checklist‐based instrument, has limitations, including the requirement for a professional evaluator and the possibility of varied responses due to evaluator differences and behavioural changes (Carrese et al., 2017). In contrast, the IDCQ is specifically designed for nurses in intensive care units and employs a self‐reporting method (Liang et al., 2022).

Upon review, the IDCQ demonstrated a high degree of cultural alignment with its intended context in Iran, surpassing other instruments. This instrument is particularly advantageous as it encompasses items deeply pertinent to Iranian society and evaluates dignity on both absolute and relative dimensions. Moreover, the concept of dignity‐based care is integral to nursing (Lin & Tsai, 2019). The nursing field melds the humanities with medical sciences (Byma & Lycette, 2023), and notions of human dignity are universally acknowledged and applied across diverse cultures (Song, 2015). The questionnaire items resonate with the philosophical tenets of nursing, especially within the Iranian nursing milieu. In the light of the lack of a specialized instrument for gauging dignified care by nurses in Iranian intensive care units, it is imperative for these professionals to systematically assess this dimension of care. Consequently, this study was undertaken to evaluate the psychometric properties of the Persian version of the IDCQ tool.

## 2 METHOD

### 2.1 Design

This methodological and psychometric study was carried out from 20 February 2022 to 19 August 2022 to assess the psychometric properties of the Persian version of the ‘IDCQ’ among Iranian nurses.

### 2.2 Participate

The study sample comprised 455 critical care nurses from six teaching hospitals in Kermanshah, selected using a stratified sampling method. From the 487 completed questionnaires, 455 were deemed suitable for analysis. For exploratory factor analysis (EFA), 150 nurses were chosen, and 305 samples were designated for confirmatory factor analysis (CFA) (Kyriazos, 2018; Lorenzo‐Seva, 2022). According to the criterion, 5–10 participants are required per item to draw sufficient inferences from EFA (Plichta & Kelvin, 2013).

Inclusion criteria included willingness and consent to participate, current employment as a nurse in intensive care units and a minimum of 2 years of work experience. If a specific questionnaire was completed less than 80%, it was excluded from the study, resulting in a selected sample of 327 nurses, and ultimately, 22 questionnaires were excluded due to lack of information, and CFA was analysed with 305 people. Therefore, at least 10 samples were selected for each item (White, 2022).

### 2.3 Study questionnaire

#### 2.3.1 Intensive Care Unit Dignified Care Questionnaire (IDCQ)

This questionnaire was developed by Liang et al. (2022) in China. It consists of two subscales: absolute dignity (9 items) and relative dignity (8 items) (17 items in total). Respondents rated each item on a 5‐point Likert scale, ranging from ‘very irrelevant’ (1 point) to ‘very relevant’ (5 points). The Cronbach's *α* coefficient for each subscale exceeded 0.9. Also, an intra‐class correlation coefficient (ICC) of 0.88 was reported after a 2‐week interval (Liang et al., 2022). A standard score of 80 or above indicates good behaviour, 60–79 suggests intermediate behaviour and below 60 denotes poor behaviour.

The developer of the questionnaire was initially contacted to conduct this study. Following cultural validation, a three‐step evaluation process was implemented as outlined by Cook and Beckman (2006) and Cook and Hatala (2016): (1) Content Evaluation to ensure comprehensive structuring of the questionnaire items. (2) Response Process to assess the alignment between the questionnaire items and the respondents' opinions and thoughts. (3) Internal Structure Evaluation to confirm the reliability of the questionnaire and the validity of the factor structure.

### 2.4 Cultural validation

The instrument underwent cultural validation using Wild et al. (2005)'s 10‐step method. The current study adopted the forward‐backward translation method. Initially, two translators independently and simultaneously translated the instrument from English to Persian. The research team reviewed and assessed these translations, combining them into a consolidated version. This consolidated version was then back‐translated into English by two different translators who were not involved in the initial translation. The research team and subject matter experts compared the back‐translated versions with the original instrument, reconciled any possible discrepancies and addressed linguistic issues. Subsequently, the final version was forwarded to the instrument developer for feedback. During the pre‐test phase, this version was distributed to several students who were requested to identify any ambiguities or possible errors. Changes were implemented based on the research team's approval. Ultimately, an expert in Persian language and literature meticulously reviewed and approved the translated version for psychometric evaluation. The final version was used for evaluation process of the psychometric properties of the tool.

#### 2.4.1 Content validity

Twelve experts and faculty members in nursing and medicine were enlisted to establish the content validity. The content validity ratio (CVR) and Content Validity Index (CVI) for each item were calculated using the Waltz and Bausell Index to assess the content validity quantitatively. Furthermore, the research team verified the qualitative content validity of the instrument (Rodrigues et al., 2017).

#### 2.4.2 Face validity

For evaluating the face validity, the final version resulting from cultural validation was given to 10 nurses (aside from the research participants), and their opinions on the clarity and comprehensibility of the items were examined and applied (Embretson, 2023).

#### 2.4.3 Multivariate normality data

The Skewness values of the items ranged from −0.74 to 2.04, and Kurtosis values from −0.605 to 1.84, both falling within the interval of (−2, 2). These values suggest that the distribution of the items is approximately symmetrical (Fonseca, 2013) (Table 2).

#### 2.4.4 Internal structure

1. Test–retest method: The instrument's reliability was assessed using the test–retest method. A group of 15 nurses completed the questionnaire simultaneously and then again 2 weeks later. The results at both times were compared with each other to evaluate the consistency of the instrument.
2. Cronbach's alpha coefficient: The Cronbach's alpha coefficient was employed to assess the internal consistency, with calculations performed for each item, each factor and the instrument as a whole.
3. Exploratory factor analysis (EFA) and confirmatory factor analysis (CFA): EFA was initially employed to validate the factor structure. Subsequently, CFA was used to validate the identified internal structure of the instrument.

### 2.5 Data analysis

This study analysed the demographic variables of the participants by calculating both absolute and relative frequencies, along with the mean and standard deviation for the model. To evaluate the instrument's reliability, internal consistency and construct validity, we employed the test–retest method, Cronbach's alpha coefficient and both exploratory and confirmatory factor analyses, respectively. Analyses were performed using SPSS version 26 and Lisrel version 8 software.

### 2.6 Ethics approval and consent to participate

Written permission was obtained from the instrument developer. The ethics committee of Kermanshah University of Medical Sciences approved the study, assigning it the ethics code: IR.KUMS.REC.1401.222. All participants provided informed written consent before their inclusion in the study. All stages of the study were explained to the participants and they were assured that their information will remain confidential and they can withdraw from the study at any time. Additionally, the study adhered to the Helsinki Declaration principles, and all methods were conducted according to relevant guidelines and regulations.

## 3 RESULTS

### 3.1 Descriptive results

In the EFA stage, a total of 150 nurses participated, with an average age of 29.01 ± 4.01, ranging from 23 to 39 years. Their average work experience was 3.72 ± 2.5, with a range of 1–14 years. During the CFA stage, 305 nurses from educational and medical centres in Kermanshah were involved, with an average age of 30.14 ± 5.01, ranging from 23 to 50 years. The average work experience among these nurses was 4.47 ± 3.76, with a range of 1–25 years. Additional demographic details are provided in Table 1.

**TABLE 1:** Demographic characteristics of participants in study.

Variables	*N* (%)	
	EFA	CFA
Gender		
Female	84 (56)	171 (56.1)
Male	66 (44)	134 (43.9)
Marital status		
Unmarried	66 (44)	145 (47.5)
Married	84 (56)	160 (5)
Grade		
Bachelor	111 (74)	243 (79.7)
Master of Science	36 (26)	62 (20.3)

### 3.2 Content and response process

The content was assessed through two distinct methods: qualitative method and quantitative method. The qualitative method involved an examination of the questionnaire items and their alignment with the study's objectives. In the quantitative method, 12 experts and faculty members contributed, yielding a content validity ratio (CVR) of 0.78, ranging from 0.53 to 1. The item‐level Content Validity Index (I‐CVI) was 0.98, falling within a range of 0.92 to 1 (Table 2).

**TABLE 2:** The ICU Dignified Care Questionnaire and results of CVI, CVR, skewness and kurtosis.

No	Items	CVI	CVR	Skewness^a^	Kurtosis^b^	*R* ^c^	_*λ*_ ^d^	*t*_value_ ^e^
1	In each work shift, I greet patients as soon as possible.	1	1	−1.56	2.03	0.538**	0.66***	12.19
2	I am polite and kind in my relationship with patients.	1	0.88	−1.84	1.68	0.516**	0.74***	14.33
3	I protect patients' privacy and avoid unnecessary physical examinations of patients.	1	0.88	−1.17	0.13	0.357**	0.53***	9.26
4	I treat patients as human beings with dignity and do not label them (e.g. I do not call them by their bed number or the name of the disease).	1	0.76	1.6	2.04	0.492**	0.58***	10.39
5	‘What kind of care would I want if I were an inpatient?’ I use this question as a guide to my nursing behaviours.	1	0.65	−0.99	0.52	0.581**	0.61***	11.09
6	I act as carefully as attainable to reduce the pain and distress of patients	0.92	0.88	−0.74	−0.74	0.586**	0.73***	14.08
7	If the patients are conscious, I explain the desired procedure to them before any nursing care.	1	0.76	−1.36	1.22	0.578**	0.69***	12.92
8	I provide nursing cares for patients with the belief that everyone is equal.	0.92	0.88	−1.54	1.96	0.459**	0.63***	11.5
9	I try to enhance the conditions around the patient, such as light and sounds.	1	0.65	−0.76	−0.35	0.536**	0.57***	10.22
10	To provide care, I collect patients' personal information from various aspects (such as education level, beliefs, customs and economic status) to give special care to each patient.	1	0.76	1.05	0.75	0.698**	0.74***	14.49
11	I set up a dynamic and adaptive nursing programme for each patient to meet the physical and psychological needs of patients according to their conditions	0.92	0.76	−0.84	0.07	0.722**	0.81***	16.35
12	I listen to the patients' and their families' requests from the bottom of my heart.	1	0.65	−0.84	0.91	0.648**	0.72***	13.9
13	I do my best to use different methods to reduce patients' and their families' anxiety, fear or other bad feelings.	1	0.76	0.85	0.73	0.643**	0.73***	14.19
14	I use special tools to quickly assess the ability of patients to make their own decisions, and I use all my efforts to maintain patients' independence in this approach	0.92	0.76	−0.43	−0.69	0.619**	0.64***	11.92
15	I involve patients' families in care (such as physical restraint programmes and early rehabilitation activities).	1	0.88	0.93	0.32	0.664**	0.70***	13.34
16	If the patients are willing to talk about themselves, I inform the PHC and their families about this interest.	1	0.53	−0.74	0.07	0.638**	0.62***	11.44
17	I always try to be patient and give patients and their families enough information about the disease to help them make the most reasonable decisions.	1	0.76	−1	1.08	0.557**	0.62***	11.52
	The ICU Dignified Care Questionnaire	0.98	0.78					

Abbreviations: CVI, Content validity ratio; CVR, Content Validity Index.

^a^ Skewness is a measure of symmetry, or more precisely, the lack of symmetry.

^b^ Kurtosis is a measure of whether the data are heavy‐tailed or light‐tailed relative to a normal distribution.

^c^ Pearson correlation coefficient.

^d^ The specific value, which is denoted by the Lambda coefficient and the statistical symbol *λ*, is calculated from the sum of the factors of the factor loads related to all the variables of that factor.

^e^ The calculated values for all factor loadings of the first and second orders are greater than 1.96 and are therefore significant at the 95% confidence level.

**p < 0.01; ***p < 0.001.

In addition, a qualitative approach was employed to evaluate the response process and to scrutinize the instrument in terms of item fluency, comprehensibility, grammatical accuracy and sentence structure. For this purpose, feedback on the instrument in these cases was solicited from 10 nurses who were not participants in the study.

### 3.3 Exploratory factor analysis (EFA)

EFA was conducted on 150 initial samples. Initially, the correlation coefficients among the questionnaire items were evaluated to ensure high correlation. The Kaiser–Meyer–Olkin (KMO) test and Bartlett's test of sphericity were employed for this assessment. The KMO test yielded a result of 0.898, and Bartlett's test of sphericity, with 136 degrees of freedom, resulted in a *χ* ^2^ value of 2442.16 and a significance level of *p* = 0.0001, justifying the application of EFA for this questionnaire.

After confirming the above assumptions, EFA was conducted on the participants' responses and the 17 questionnaire items. The maximum likelihood method (ML) and oblique promax rotation were applied for factor extraction. Table S1 presents the extracted communalities for each item using the principal component analysis (PCA) method, along with the results of the corresponding stability tests. Factors with an eigenvalue greater than one were considered to ascertain the number of factors. The preliminary analysis suggested that two factors or components were appropriate for further analysis. Table S2 displays the extracted factors, their eigenvalues, the percentage contribution of each factor in explaining the variance of the 17 items and the cumulative variance explained by each of the two factors (66.67%).

**FIGURE 1:**
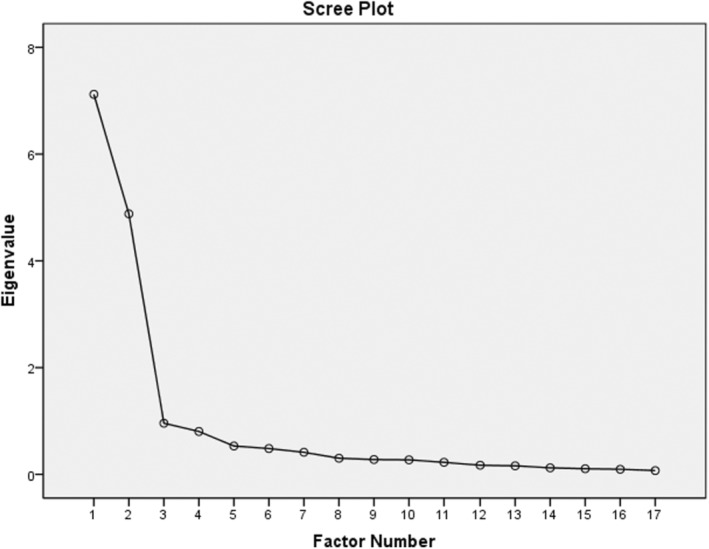
Scree plot of the extracted elements of the questionnaire.

In summary, the two factors with eigenvalues exceeding one explained 66.67% of the variance across the 17 items. The first factor contributed 39.49%, while the second accounted for 27.17% of the cumulative variance, as illustrated in Figure 1. Items with a factor loading greater than 0.3 and possessing the highest loadings were assigned to the appropriate component (Table S3).

### 3.4 Confirmatory factor analysis (CFA)

CFA was conducted on 305 samples. The CFA sought to evaluate the compatibility of the predefined factor model with the observed data. Before the analysis, it was established that the data exhibited multivariate normality, as indicated by the skewness and kurtosis values. Furthermore, the factor loadings for the questionnaire items were all above 3.29, denoting a significance level of 0.001 (Table 2).

**FIGURE 2:**
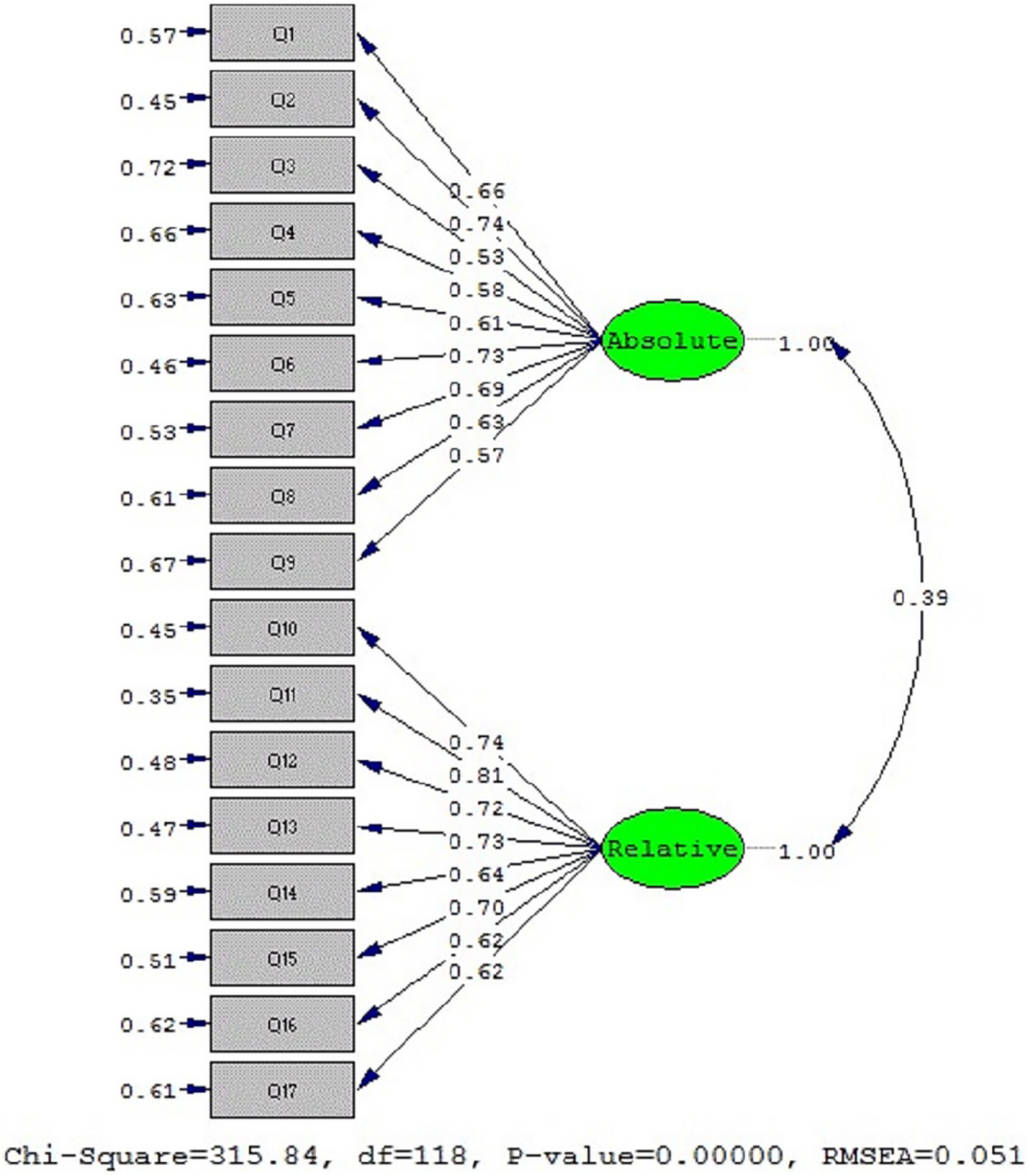
Two‐section model of the ICU Dignified Care Questionnaire in Iranian nurses (standard state).

Confirmatory factor analysis (CFA) was executed on two factors comprising a total of 17 items. Figure 2 illustrates the CFA results, detailing the significance (A) and standardization (B) of the coefficients. Given that all values exceeded the critical threshold of 1.96, there was no need to eliminate any item. Additionally, the CFA results indicated that the IDCQ model possesses satisfactory fit indices, with an RMSEA value of 0.51 and an SRMR value of 0.46 (Table 3).

**TABLE 3:** Fit indicators confirmatory factor analysis Persian ICU Dignified Care Questionnaire and its sections.

Fit indicators	Criterion	Level	Interpretation
*χ* ^2^/df	≤3	2.67	Optimal fit
df		118	
*χ* ^2^		315.84	
*p*_value_		0.0001	
NNFI (or TLI)	>0.9	0.92	Optimal fit
CFI^4^	>0.9	0.93	Optimal fit
AGFI^5^	>0.8	0.861	Optimal fit
SRMR	<0.05	0.046	Optimal fit
RMSEA	0.05–0.08	0.051	Optimal fit

### 3.5 Correlation between factors and instrument's reliability

Pearson's correlation test revealed a significant and direct correlation (*p* < 0.001) between the IDCQ subscales and the total scale score. To evaluate the internal reliability (Internal validity) of the IDCQ, a Cronbach's *α* coefficient of 0.887 for the entire scale was computed. The reliability coefficients were 0.953 for the absolute dignity factor and 0.93 for the relative dignity factor, demonstrating that the subscales possess the requisite reliability for measurement (Table 4). Additionally, a test–retest correlation coefficient of 0.875 was observed over a 1‐week interval.

**TABLE 4:** Internal consistency of the ICU Dignified Care Questionnaire and its factors in nursing staffs.

Factors/section and scale	Items No.	Item‐total correlation	Cronbach's *α*	
Absolute dignity	1	0.615	0.872	0.855
	2	0.653	0.873	
	3	0.582	0.877	
	4	0.576	0.874	
	5	0.550	0.871	
	6	0.580	0.871	
	7	0.555	0.871	
	8	0.479	0.875	
	9	0.473	0.873	
Relative dignity	10	0.456	0.867	0.86
	11	0.295	0.864	
	12	0.428	0.868	
	13	0.510	0.868	
	14	0.535	0.869	
	15	0.518	0.868	
	16	0.392	0.869	
	17	0.458	0.872	
ICU Dignified Care Questionnaire		0.877		

## 4 DISCUSSION

This study has confirmed the validity and reliability of the Persian version of the IDCQ in assessing dignity‐based care among Iranian nurses. The CVI and CVR values, which were determined to be 0.98 and 0.78, respectively, align with the results of previous studies (Beach et al., 2018; Zamanzadeh et al., 2015). The KMO value of 0.898 indicates the data's suitability for factor analysis. Liang et al. (2022)'s study utilized oblique promax factor analysis, which yielded two distinct factors and a slightly higher KMO value. This study employed a distinct methodology, including model fit, validity and data normality criteria, to ascertain the alignment of results and all items in the Persian version of the IDCQ. These findings indicate that the concepts of human dignity and its related factors are applicable cross‐culturally.

Two components of dignity, both absolute and relative, were validated by exploratory factor analysis (EFA) in the Persian version of the IDCQ within the Iranian nursing community. The EFA results indicated that these factors accounted for 70.59% of the item variances. In related research, Liang et al. (2022)'s study demonstrated that the same two components explained 62.8% of the variance. Consequently, the Persian IDCQ successfully identified two components of dignity, accounting for 70.59% of item variances.

Liang et al. (2022)'s study yielded similar results. Lin and Tsai (2019) identified both absolute and relative elements of dignity, proposing six variables. Kadivar et al. (2018) and Nouri et al. (2022) highlighted the roles of beliefs, societal influences and varying roles in the definition of dignity. This study considers the unique factors of the community and cultural nuances, demonstrating their impact on dignity. Although the identified factors are consistent with shared concepts across communities in various studies, differences are observed in research exploring distinct concepts and communities.

Some studies, such as Carrese et al. (2017), concentrated on developing a checklist to evaluate dignity and respect in ICU patients, identifying only one factor. However, ratings for dignity and respect varied among different ICU units, with specialized staff receiving higher scores. These variations may be attributed to professional standards, cultural differences in care and sample size.

The literature review revealed that the validity and reliability of various instruments related to patient dignity are remarkably consistent across different societies and cultures (Elf et al., 2017; Mikuletič et al., 2024). This uniformity may stem from the universal moral principles of intrinsic dignity and respect for humanity (Harstäde et al., 2018). Human dignity is acknowledged as a core principle of nursing, as underscored by the International Council of Nurses (Stikholmen et al., 2024). Nursing is fundamentally based on respect for human rights, particularly cultural rights, and the right to be treated with dignity and respect (Stephen Ekpenyong et al., 2021). Nurses play a crucial role in upholding human dignity in their interactions with patients and within healthcare teams. Nevertheless, the challenges presented by automated medicine have accentuated the significance of this aspect of care (Peyvakht et al., 2020). Consequently, the education of healthcare workers and the development of culturally sensitive instruments for assessing patient dignity have become imperative (Kaihlanen et al., 2019; Lee et al., 2020).

Despite varying factor analysis rotation methods, the overall index of Cronbach's *α* was 0.887, in line with Liang et al. (2022)'s findings. This finding is corroborated by other studies (Lin & Tsai, 2019; Nouri et al., 2022). The established reliability of this questionnaire underscores its utility as an instrument for measuring dignity‐based care in ICUs, highlighting the critical importance of preserving patient dignity and respect.

### 4.1 Limitations and study strengths

The current study was centred on the community of intensive care nurses in the city of Kermanshah, located in Western Iran. It is important to note that the research took place during the concluding phase of the COVID‐19 pandemic, which could have influenced the findings. The challenges faced by ICU nurses in delivering care amidst the pandemic led to the adoption of an electronic questionnaire.

There is a possibility that the questionnaire might have been completed by individuals other than nurses, such as family members. Despite the cultural differences across various geographical areas of Iran, it may be feasible to explore the application of this instrument in other regions. Furthermore, the results could have broad relevance throughout different parts of Iran, as most regions maintain consistent professional and routine standards of care.

### 4.2 Implications for nursing practice

Critically ill patients hospitalized in ICUs are highly vulnerable, and dignity is of paramount importance to them. The loss of dignity is a common occurrence in the intensive care unit (ICU). Nurses play an essential role in promoting dignity among hospitalized patients in these units. The results of this study indicate that the Persian version of the IDCQ possesses acceptable validity and reliability. Hence, this instrument can serve as a guide in nursing practice to assist Iranian nurses working in ICUs in self‐assessing their behaviours to preserve the patient's dignity. Additionally, the IDCQ can be instrumental in developing the content of intervention programmes and evaluating the effectiveness of such programmes in enhancing the dignity of ICU patients.

## 5 CONCLUSION

The Persian version of the IDCQ, as applied in Iranian intensive care units, comprises 17 items and two factors: absolute dignity and relative dignity. The IDCQ, noted for its high validity and reliability, enables ICU nurses to evaluate how they uphold dignity in their work. Its capacity to pinpoint areas of concern can help to enhance the quality of dignified care. Nursing managers can employ the IDCQ to develop practices that foster dignified care at the organizational level and to evaluate the quality of humanistic care provided in the ICU.

## AUTHOR CONTRIBUTIONS

All authors participated and approved the study design. Khalil Moradi and Amir Jalali contributed to designing the study, Niloufar Darvishi; Parnia Kalhory; Salam Vatandost and Fateme Merati collected the data, and data analyses were done by Amir Jalali. The final report and article were written by Amir Jalali; Khalil Moradi; Niloufar Darvishi; Salam Vatandost; Parnia Kalhory and Fateme Merati; and all authors read and approved the final manuscript.

## FUNDING INFORMATION

The Kermanshah University of Medical Sciences funded the research project. The funds were allocated toward the study's design and data collection.

## CONFLICT OF INTEREST STATEMENT

The authors declare no conflicts of interest.

## ETHICS STATEMENT

A written permission was secured from the developer of scale and the ethic committee of the Kermanshah University of Medical Sciences approved the study under the ethic code: IR.KUMS.REC.1401.222.

## CONSENT TO PARTICIPATE

All participants completed informed written consent to participate in the study. In addition, the principles of Helsinki Declaration were observed. All methods were performed per the relevant guidelines and regulations.

## Supporting information


Tables S1–S3

## Data Availability

The data sets used in the study are available from the corresponding author on reasonable request.
