# Supplementary material for: Intensive care unit dignified care: Persian translation and psychometric evaluation
Source: Nurs Open. 2024 Jul 8;11(7):e2238. doi: 10.1002/nop2.2238 (PMC11231042; doi:10.1002/nop2.2238)
Supplement: Supplementary file 1 — Tables S1–S3 [file NOP2-11-e2238-s001.docx]

**Supplementary table 1**. Subscription values for each item and stability test

| items | Extraction | Corrected Item-Total Correlation | Cronbach's Alpha if Item Deleted |
| --- | --- | --- | --- |
| Q1 | .568 | .516 | .897 |
| Q2 | .661 | .541 | .896 |
| Q3 | .351 | .300 | .902 |
| Q4 | .613 | .532 | .897 |
| Q5 | .760 | .551 | .895 |
| Q6 | .900 | .577 | .895 |
| Q7 | .829 | .590 | .895 |
| Q8 | .782 | .526 | .896 |
| Q9 | .780 | .544 | .896 |
| Q10 | .803 | .697 | .890 |
| Q11 | .755 | .696 | .890 |
| Q12 | .671 | .678 | .891 |
| Q13 | .812 | .680 | .891 |
| Q14 | .336 | .456 | .899 |
| Q15 | .776 | .651 | .892 |
| Q16 | .388 | .508 | .898 |
| Q17 | .551 | .567 | .895 |

**Supplementary table2**: Variance percentage and specific values of different factors

| Factor | Initial Eigenvalues | | | Extraction Sums of Squared Loadings | | | Rotation Sums of Squared Loadings |
| --- | --- | --- | --- | --- | --- | --- | --- |
|  | Total | % of Variance | Cumulative % | Total | % of Variance | Cumulative % | Total |
| 1 | 7.120 | 41.882 | 41.882 | 6.714 | 39.496 | 39.496 | 6.373 |
| 2 | 4.880 | 28.708 | 70.590 | 4.620 | 27.177 | 66.673 | 5.297 |
| 3 | .959 | 5.639 | 76.229 |  |  |  |  |
| 4 | .804 | 4.731 | 80.961 |  |  |  |  |
| 5 | .532 | 3.128 | 84.089 |  |  |  |  |
| 6 | .484 | 2.849 | 86.938 |  |  |  |  |
| 7 | .414 | 2.434 | 89.371 |  |  |  |  |
| 8 | .302 | 1.777 | 91.148 |  |  |  |  |
| 9 | .279 | 1.639 | 92.787 |  |  |  |  |
| 10 | .273 | 1.604 | 94.392 |  |  |  |  |
| 11 | .226 | 1.331 | 95.723 |  |  |  |  |
| 12 | .172 | 1.010 | 96.733 |  |  |  |  |
| 13 | .160 | .940 | 97.673 |  |  |  |  |
| 14 | .123 | .723 | 98.396 |  |  |  |  |
| 15 | .106 | .621 | 99.017 |  |  |  |  |
| 16 | .096 | .564 | 99.581 |  |  |  |  |
| 17 | .071 | .419 | 100.000 |  |  |  |  |

Supplementary table3. : Matrix of factor loadings of questionnaire questions on components after rotation

| Rotated Component Matrix^a^ | | |
| --- | --- | --- |
|  | Component | |
|  | 1 | 2 |
| Q1 | .749 | .026 |
| Q2 | .812 | .006 |
| Q3 | .601 | -.128 |
| Q4 | .781 | .011 |
| Q5 | .867 | .022 |
| Q6 | .950 | -.007 |
| Q7 | .900 | .053 |
| Q8 | .886 | -.014 |
| Q9 | .881 | .013 |
| Q10 | -.022 | .900 |
| Q11 | .012 | .867 |
| Q12 | .046 | .810 |
| Q13 | -.025 | .905 |
| Q14 | .030 | .574 |
| Q15 | -.046 | .888 |
| Q16 | .026 | .618 |
| Q17 | -.036 | .747 |
| Extraction Method: Maximum Likelihood. | | |
| Rotation Method: Promax with Kaiser Normalization. | | |
